# Fast and selective cell isolation from blood sample by microfiber fabric system with vacuum aspiration

**DOI:** 10.1080/14686996.2016.1243006

**Published:** 2016-11-25

**Authors:** Takayuki Ueki, Akifumi Yoshihara, Yuji Teramura, Madoka Takai

**Affiliations:** ^a^ Department of Bioengineering, The University of Tokyo, Tokyo, Japan; ^b^ Department of Immunology, Genetics and Pathology (IGP), Uppsala University, Uppsala, Sweden

**Keywords:** Circulating tumor cells (CTCs), electrospun microfiber fabric, EpCAM, vacuum aspiration, 30 Bio-inspired and biomedical materials, 102 Porous / Nanoporous / Nanostructured materials, 212 Surface and interfaces, 211 Scaffold / Tissue engineering/Drug delivery

## Abstract

Since circulating tumor cells (CTCs) are tumor cells which are found in the blood of cancer patients, CTCs are potential tumor markers, so a rapid isolation of CTCs is desirable for clinical applications. In this paper, a three-dimensional polystyrene (PS) microfiber fabric with vacuum aspiration system was developed for capturing CTCs within a short time. Various microfiber fabrics with different diameters were prepared by the electrospinning method and optimized for contact frequency with cells. Vacuum aspiration utilizing these microfiber fabrics could filter all cells within seconds without mechanical damage. The microfiber fabric with immobilized anti-EpCAM antibodies was able to specifically capture MCF-7 cells that express EpCAM on their surfaces. The specificity of the system was confirmed by monitoring the ability to isolate MCF-7 cells from a mixture containing CCRF-CEM cells that do not express EpCAM. Furthermore, the selective capture ability of the microfiber was retained even when the microfiber was exposed to the whole blood of pigs spiked with MCF-7 cells. The specific cell capture ratio of the vacuum aspiration system utilizing microfiber fabric could be improved by increasing the thickness of the microfiber fabric through electrospinning time.

## Graphical abstract

**Figure:**
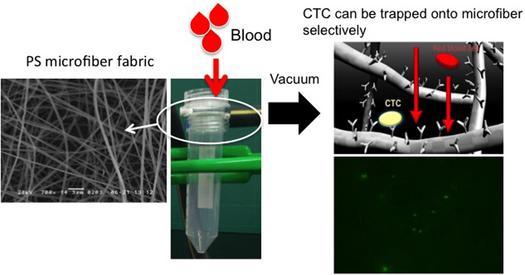

## 1. Introduction

Circulating tumor cells (CTCs) are rare tumor cells which are found in bloodstreams of cancer patients.[1,2] CTCs have gained much attention as potential tumor markers because there is a strong correlation between the number of CTCs and the progression of cancer.[3–5] Moreover, molecular analysis of CTCs provides useful information for cancer diagnosis since CTC-derived gene mutation has been reported.[6,7] However, since the number of CTCs is estimated to range between 1 and 100 cells ml^–1^ of whole blood,[8,9] it has been difficult to efficiently collect CTCs from the blood of patients. One of the approaches to capture CTCs is the use of an antibody that is specific for epithelial cell adhesion molecule (EpCAM) expressed on CTCs, but not on blood cells. For example, EpCAM was highly expressed on CTCs derived from prostate and breast tumors.[10–12] Some papers reported that the anti-EpCAM-antibody-immobilized microchips could capture the CTCs with high efficiency.[13–15] Although CTCs have been captured on microchips successfully, these systems are still not practical for clinical diagnosis, particularly for point-of-care testing. This is because microchips need external equipment such as tubes and well-controlled pumps, and the system requires experts to be correctly handled. In addition, because sufficient number of CTCs must be captured for extracting genes, it will take several hours using these microchip systems to capture CTCs from 10 ml of whole blood when the flow rate is 1–2 ml h^–1^. Therefore, there is a need for a more rapid and simple device for capturing CTCs for clinical use.

Another approach is size separation of CTCs by microchannel,[16–18] microfiltration system,[19] and acoustic separation.[20] Zheng et al*.* [20] reported a device that can enrich the CTCs from whole blood by size separation when the CTCs are significantly larger than blood cells^[20]^. They could capture CTCs from 10-fold-diluted whole blood within 3–5 min with high efficiency without the use of antibodies. However, the size of CTCs may be different among patients, and this may result in false negative diagnosis if the smaller CTCs fail to be captured.

**Figure 1.**
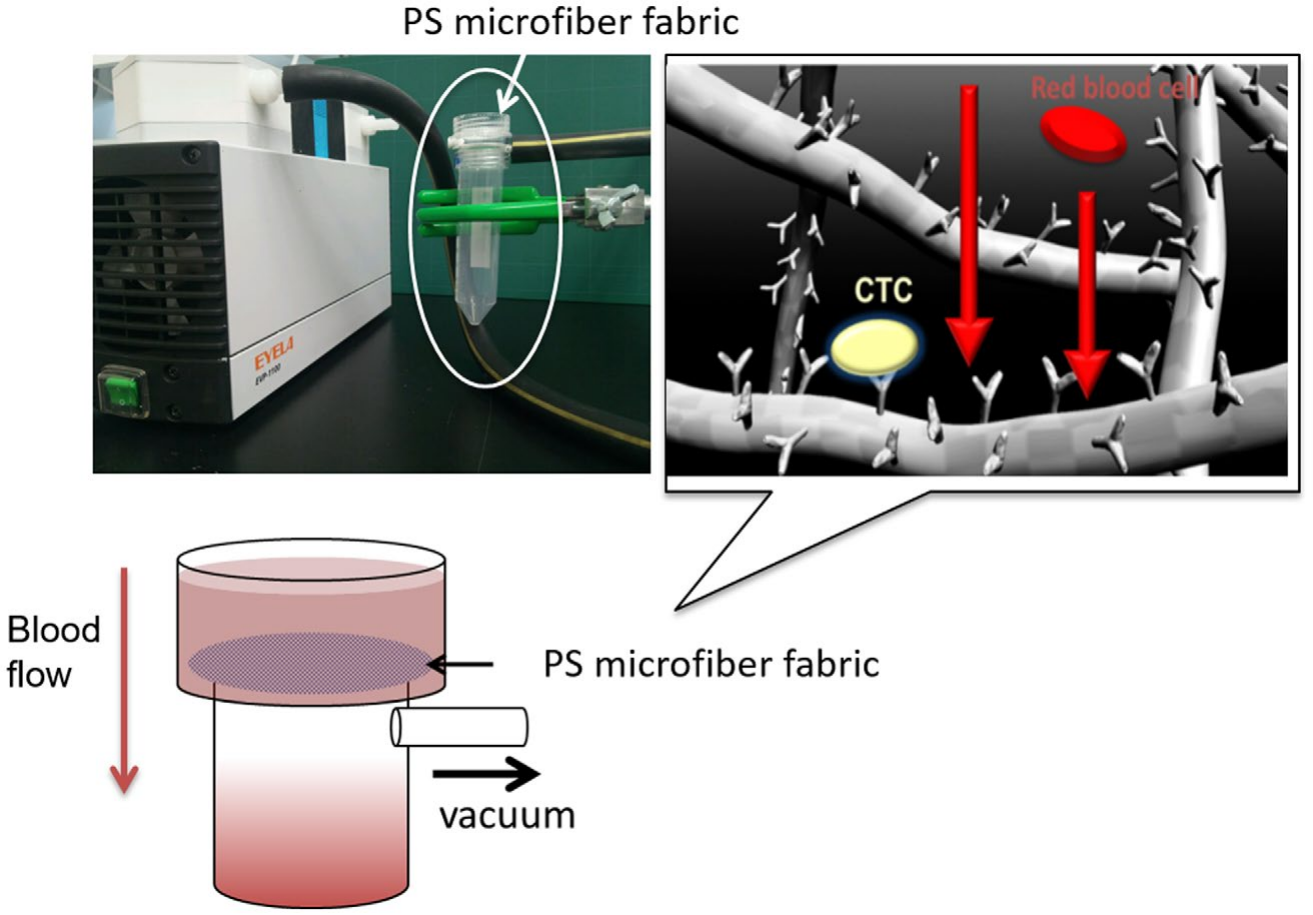
The setup of the specific cell capturing microfiber system with a vacuum pump. Cell suspension is passed through the microfiber fabric by vacuum pumping. All cells flow out through the micropores of the three-dimensional microfiber fabric. The flow rate was approximately 10 ml min^–1^.

To overcome these problems, in this study we aimed to fabricate a microfiber fabric system with vacuum aspiration and immobilized anti-EpCAM antibodies. Here, we used three-dimensional polystyrene (PS) microfiber fabricated by an electrospinning method, and the pore size and thickness were controlled for improving the efficiency of cell capturing from blood samples. The system could rapidly isolate MCF-7 cells, which were used as a model of CTCs, from whole blood and around 10 ml of whole blood could filter through in several seconds (Figure 1). Also, it can be converted to miniaturized cell capturing systems that can be used as point of care testing devices.

## 2. Experimental

### 2.1. Materials

Polystyrene pellets (SGP 10) were obtained from PS Japan Co. (Tokyo, Japan). Tetrahydrofuran and *N*,*N*′-dimethylformamide were obtained from Kanto Chemical Co. (Tokyo, Japan). Fetal bovine serum (FBS, qualified, heat-inactivated, USDA-approved), penicillin-streptomycin (liquid), Dulbecco’s phosphate-buffered saline (10×, no calcium, no magnesium), Roswell Park Memorial Institute medium 1640 (1×), Dulbecco’s modified Eagle medium (DMEM, 1×, high glucose), Celltracker Green, Celltracker Orange and 0.25% Trypsin-ethylenediaminetetraacetic acid (EDTA, 1×), Phenol Red were obtained from Thermo Fisher Scientific K.K. Inc. (Waltham, MA, USA). Albumin from bovine serum and Triton^®^ X-100 were obtained from Sigma-Aldrich Corp. (St Louis, MO, USA). Aluminum mesh was obtained from Kurebaa Inc. (Aichi, Japan). Human EpCAM, TROP-1 antibody (polyclonal goat IgG) was obtained from R&D system Inc. (Minneapolis, MN, USA). Whole blood from pig was generously supplied by Gunma Meat Wholesale Market Co., Ltd (Gunma, Japan).

### 2.2. Fabrication of electrospun PS microfiber fabric

PS microfiber fabric was fabricated by electrospinning (Sprayer ES-1000, Fuence Co., Ltd, Saitama, Japan). Polystyrene pellets (Mw = 9.0 × 10^5^) were dissolved in a mixed solvent of tetrahydrofuran and *N*,*N*′-dimethylformamide (1:1, by volume) with Triton-X (0.5 wt%). The PS solutions with various concentrations (5, 10, 15, 20 and 25 wt%) were placed in a syringe fitted with a needle and the feeding rate of the PS solution was 0.9 ml h^–1^. The microfiber was collected onto the rotating drum (600 rpm) covered with aluminum mesh (wire diameter = 0.10 mm, aperture = 0.154 mm, aperture ratio = 36.8%) as a support substrate of microfiber. The PS solution was sprayed at applied voltage of 20 kV while the collector was grounded. The distance between the collector and the syringe was 100 mm. The thickness of microfibers was dependent on electrospinning time. The electrospun PS microfiber fabrics on aluminum foil were observed by scanning electron microscopy (SEM, SM-200, Topcon, Tokyo, Japan). All samples for SEM observations were coated with 10 nm gold by using an ion coater (Quick Auto Coater SC-701AT, Sanyu Electron Co., Tokyo, Japan).

### 2.3. Hemocompatibility assay for electrospun PS microfiber fabric with whole blood

In order to examine the hemocompatibility of electrospun PS microfiber fabrics with different microstructure, whole blood mixed with heparin was flowed into the PS microfiber fabrics by a diaphragm pump (LABOPORT Oil-Free Diaphragm Pumps, KNF Neuberger Inc., Trenton, NJ, USA, ultimate pressure; 68 Torr) and we evaluated the degree of hemolysis. The blood was passed through the fabric within 20 s. The porosity of the microfiber fabrics was 53.7%, which was calculated from 3D image by confocal laser scanning microscopy (LSM510, Carl Zeiss Microscopy Co., Ltd, Jena, Germany). The microfiber fabrics were fluorescently observed after adsorption of fluorescein isothiocyanate-bovine serum albumin (FITC-BSA, 1 mg ml^–1^, Sigma-Aldrich Corp.). As a positive control, the whole blood collected from young goat (Japanese-Saanen or hybrid of Japanese-Saanen, Female, Merryland Co., Ltd, Nagano, Japan) was lysed with VersaLyse™ (Beckman Coulter, Pasadena, CA, USA). The whole blood (1 ml) and VersaLyse™ (10 ml) were mixed for 10 min at room temperature. The collected whole bloods passing through the electrospun PS fabrics with a vacuum aspiration system (Figure 1) were centrifuged at 300 rpm for 10 min. The absorbance at 560 nm of the collected supernatant was measured with a plate reader (ARVO sx, Perkin Elmer Co., Waltham, MA, USA).

### 2.4. Cell culture and live cell labeling

Human breast cancer epithelial MCF-7 cells (American Type Culture Collection, ATCC; Manassas, VA, USA) were cultured in DMEM supplemented with 10% FBS, 10 μg ml^–1^ insulin, non-essential amino acids (NEAA), 1 mM sodium pyruvate, 50 U ml^–1^ penicillin, and 50 μg ml^–1^ streptomycin. Human cervical cancer cells, HeLa cells (RIKEN BRC, Tsukuba, Japan) were cultured in DMEM supplemented with 10% FBS, 50 U ml^–1^ penicillin, and 50 μg ml^–1^ streptomycin. CCRF-CEM cell line, established from acute lymphoblastic leukemia T cells, was obtained from the Health Science Research Resources Bank (Tokyo, Japan). Suspended CCRF-CEM cells were cultured in RPMI-1640 medium supplemented with 10% FBS, 50 U ml^–1^ penicillin and 50 μg ml^–1^ streptomycin. All cells were cultured at 37 °C with 5% CO_2_ and 95% air. Adherent cells were harvested using 0.05% trypsin-EDTA. Each cell line was labelled with Celltracker Green or Celltracker Orange following the manufacturer’s instructions.

### 2.5. Immobilization of antibody on electrospun PS microfiber fabric

To immobilize the antibody on PS microfiber fabric, we conducted the pretreatment of the electrospun PS microfiber fabrics with hydrophobicity because of the difficulty in permeability. The PS microfiber fabrics were immersed in ethanol for 10 s and then ethanol was replaced with pure water for 10 s. Then, the electrospun PS microfiber fabric was immersed into 10 μg ml^–1^ anti-EpCAM antibody solution (phosphate buffered saline, PBS pH 7.4) for 1 h. The PS microfiber fabric was washed with PBS (pH 7.4). Finally, for the blocking treatment, the anti-EpCAM antibody-immobilized electrospun PS microfiber fabric was incubated in 1 wt% BSA solution (PBS, pH 7.4) for 1 h, and washed with PBS.

### 2.6. Capturing of cells on anti-EpCAM antibody-immobilized electrospun PS microfiber fabric

All cell lines (MCF-7, HeLa and CCRF-CEM) were stained with Celltracker Green or Celltracker Orange before use in this experiment. Cell suspension of MCF-7 or HeLa (1.0 × 10^5^ cells ml^–1^ in 4 ml of culture medium with FBS) was applied and passed within 20 s by a vacuum pump (ultimate pressure; 68 Torr). Then, the PS microfiber fabric was washed with PBS. The PS microfiber fabric was observed by upright fluorescence microscope (Axioskop 2plus, Carl Zeiss). Also, a mixed cell suspension of MCF-7 and CCRF-CEM was applied to the electrospun PS microfiber fabric. The CCRF-CEM suspension (1.0 × 10^5^ cells in 2 ml) was mixed with MCF-7 suspension with different cell concentrations (1.0 × 10^5^, 1.0 × 10^4^, and 1.0 × 10^3^ cells in 2 ml). The mixed cell suspension was passed through the PS microfiber fabric. After washing with PBS, the PS microfiber fabric was observed by upright fluorescence microscope.

In order to examine the capturing ability in whole blood, we used whole pig blood. MCF-7 (1.0 × 10^3^ cells) was mixed with 10 ml of whole pig blood (hybrid of Yorkshire, Landrace and Duroc, six months old, male, Gunma Meat Wholesale Market Co., Ltd). The mixed blood was passed through the fabric within 20 s. After washing with PBS, the PS microfiber fabric was observed by an upright fluorescence microscope. We used WST (water soluble tetrazolium salts) assay to count the cells before and after the cells were applied to the PS microfiber fabric. The WST method is a cell viability assay method that can count the number of cells by enzyme activity using the 2-(2-methoxy-4-nitrophenyl)-3-(4-nitrophenyl)-5-(2,4-disulfophenyl)-2*H*-tetrazolium (WST-8). The absorbance at 450 nm was measured after 2 h incubation at 37 °C using a plate reader (Appliskan 5230020, Thermo Electron Corporation, Waltham, MA, USA).

## 3. Results and discussion

### 3.1. Microstructure of electrospun microfiber PS fiber

**Figure 2.**
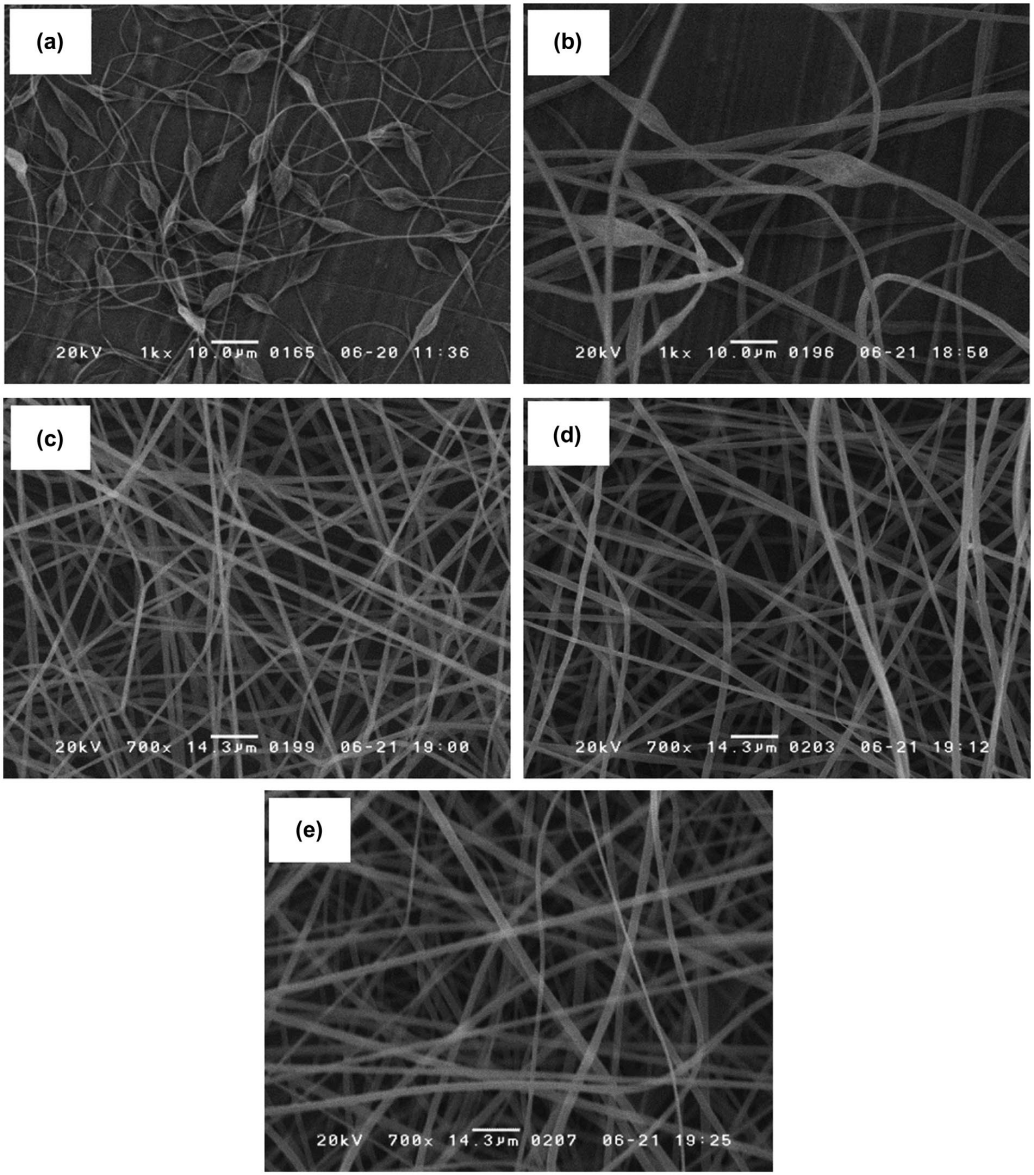
SEM images of PS microfibers fabricated using different concentrations of PS solution: (a) 5, (b) 10, (c) 15, (d) 20 and (e) 25 w/v%.

Electrospun PS microfiber fabric was fabricated with the electrospinning method. First, we optimized the electrospun PS microfiber fabric with different concentrations of PS solution through observation with SEM as shown in Figure 2(a–e). Non-uniform microstructure including microfiber and microclumps was observed on the electrospun PS microfiber when 5 and 10 w/v% PS solution was used. On the other hand, well-organized microfiber structure was observed when 15, 20 and 25 w/v% PS solutions were used for electrospinning. Also, the diameter of the microfiber increased with the increase of PS concentration (15 w/v%: 1.4 ± 0.1 μm, 20 w/v%: 1.9 ± 0.1 μm, 25 w/v%: 2.2 ± 0.1 μm). There was a high correlation between the pore size and PS concentration. The pore size in diameter was 10 ± 6, 10 ± 6 and 13 ± 8 μm, for 15, 20, and 25 w/v%, respectively. The size of CTCs and leukocytes are approximately 10 μm, which is equivalent to the pore size of those microfiber fabrics. For efficiency of cell capturing, the total contact area of the microfiber to blood sample is another important factor. Considering the surface area per volume, electrospun PS microfiber fabric fabricated with 20 w/v% had the largest contact surface area with a suitable pore size, so we decided to use the electrospun PS microfiber fabric for the following experiments. The thickness of electrospun PS microfiber fabric could be controlled by the electrospinning time using 20 w/v% PS solutions. The thickness linearly increased up to approximately 100 μm with electrospinning time in 4 min, suggesting a strong correlation between the electrospinning time and the thickness. However, when the electrospinning time was increased to over 4 min, it was impossible to control the uniform thickness of PS microfiber fabric due to the concentration of applied voltage on insulated microfiber fabric of the rotating drum.[21] Therefore we used the electrospun PS microfiber fabric under 100 μm of thickness.

### 3.2. Evaluation of fabricated electrospun PS microfiber fabric

**Figure 3.**
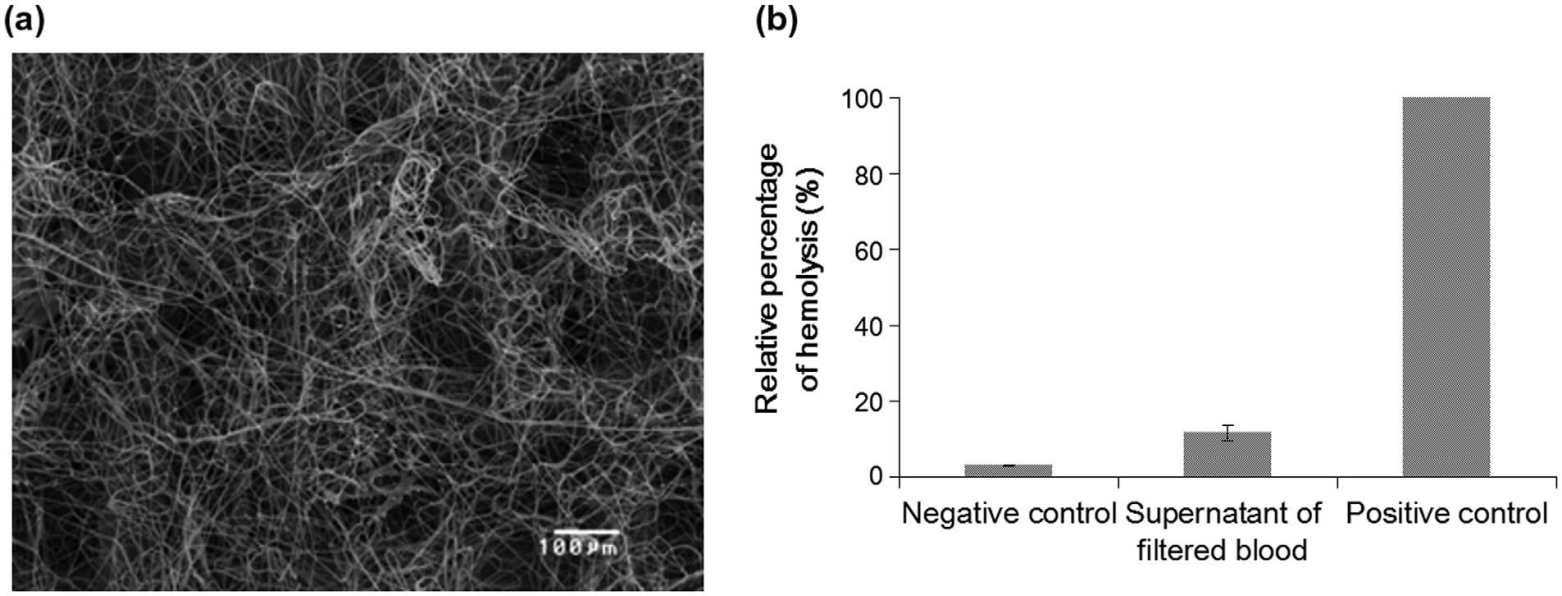
Hemocompatibility assay with whole blood. (a) SEM image of the PS microfiber after vacuum aspiration of whole blood is shown. Flow rate of whole blood was 1.8 ml min^–1^. (b) The result of hemolytic assay is shown. The whole blood before and after the experiment was centrifuged to collect the supernatant. The absorbance at 560 nm was measured to calculate the percentage of hemolysis.

In order to examine the influence on the microstructure after the passage of whole blood with the vacuum pump, the electrospun PS microfiber fabric was observed by SEM (Figure 3(a)). Since the electrospun PS microfiber fabric is flexible because of high porosity, there was no damage, e.g. cleavage of microfibers, after the passage of whole blood. This indicated that our electrospun PS microfiber fabric was strong enough for vacuum aspiration of whole blood. In addition, no trapping of blood cells was observed in the three dimensional microstructure of the electrospun PS microfiber fabric. Basically, all blood cells are passed through the microfiber fabric by the aspiration, so blood cells were not entrapped in the microfiber fabric. Also, the pore size of the fabric is optimized so that blood cells are not entrapped.

The influence of vacuum pressure on cells was also examined. The hemolysis of erythrocytes was calculated before and after blood vacuuming (Figure 3(b)). Fresh whole blood was used as negative control, and hemolyzed whole blood was used as positive control. The ratio of hemolysis was less than 10% after blood vacuuming. Although there was slight hemolysis, most cells were still intact. The PS microfiber fabric had a suitable mechanical strength, an excellent permeable property of whole blood by vacuuming and furthermore, the system did little damage to cells in the blood. Also, it was suggested that both the pore size of our PS microfiber fabric and vacuum pressure were appropriate for less hemolysis.[22]

### 3.3. Evaluation of cell capturing ability

**Figure 4.**
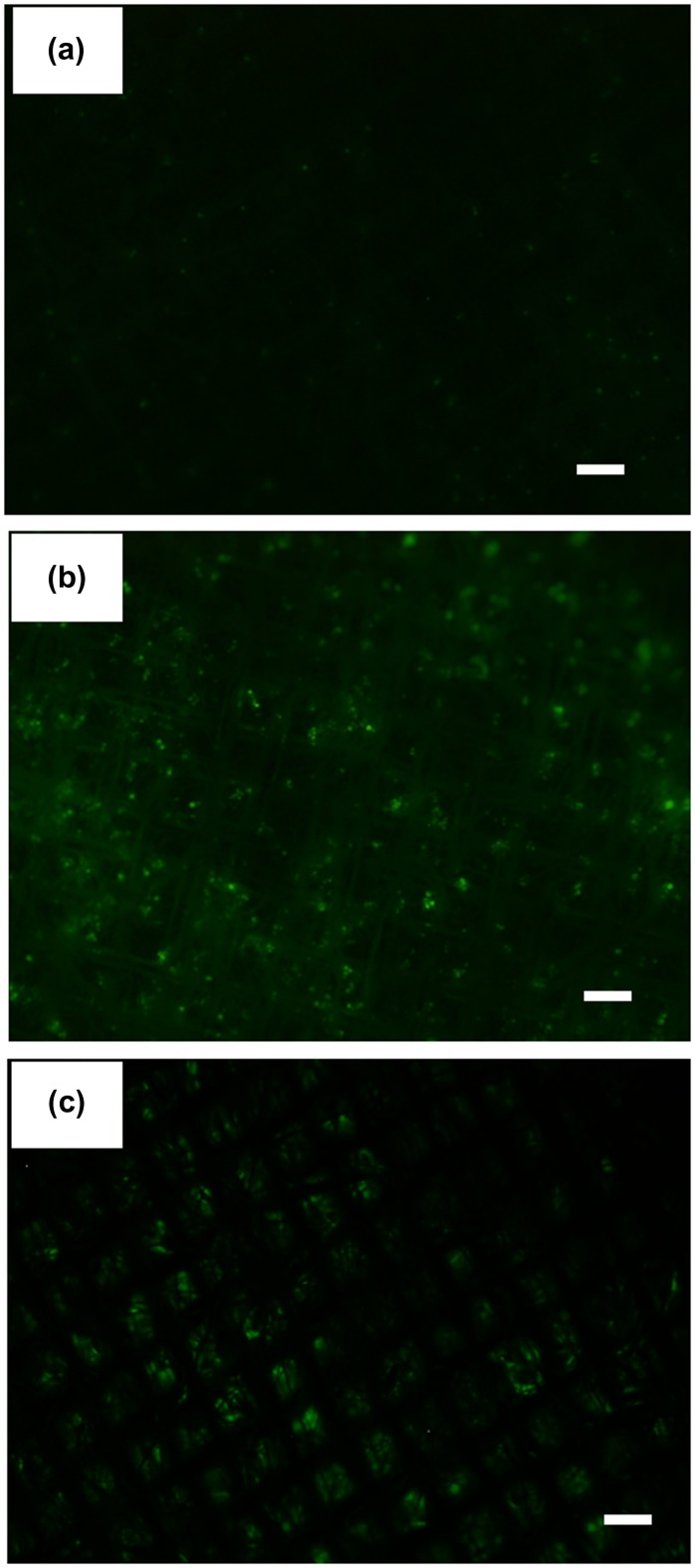
Fluorescence images of PS microfiber fabric on aluminum mesh with captured cells. (a) HeLa, (b) MCF-7 cells and (c) EpCAM-blocked MCF-7 cells by antibody were separately passed through the microfiber fabric. Here, the autofluorescence from PS microfiber was slightly observed. Scale bars: 250 μm.

Anti-EpCAM antibody was used to capture MCF-7 cells as a model of CTCs, which express EpCAM on their cell membrane. First, we examined the capturing ability of the electrospun PS microfiber fabric where anti-EpCAM antibody was immobilized using two kinds of cancer cell lines, MCF-7 and HeLa. We have previously reported that the electrospun PS microfiber fabric with antibody immobilization has an ability to capture the specific antigen rapidly with vacuuming.[23] The mechanism of the rapid assay is an increase of the reaction probability by flow-through of liquid samples through the microfiber fabrics using vacuum aspiration system. So, we expected that specific cells could be captured on the microfiber fabric in short time in a similar manner using the same principle. It is known that MCF-7 cells express EpCAM and HeLa cells do not. Here these cell types were used and fluorescently labeled in advance. When MCF-7 or HeLa cells were filtered with 10 ml min^–1^ flow rate to the anti-EpCAM-antibody-immobilized electrospun PS microfiber fabric under vacuum, the fluorescently labeled MCF-7 cells were observed on the microfiber fabric while the fluorescently labeled HeLa cells were not (Figure 4(a) and (b)). In addition, when MCF-7 cells were treated with anti-EpCAM antibody in advance, there was no MCF-7 observed on the microfiber fabric (Figure 4(c)). Here, the lack in sharpness in the images was caused by the three-dimensional microstructure of the microfiber fabric, and thus the cells were captured not only at the top surface but also inside the fabric. These results indicated that the capture of MCF-7 cells was specifically through the interaction between EpCAM on MCF-7 cells and the anti-EpCAM antibody on the PS microfibers. The cell capturing process was performed at high flow rate (10–20 ml min^–1^) compared to the microchip system where the flow rate was generally 1–2 ml h^–1^.

**Figure 5.**
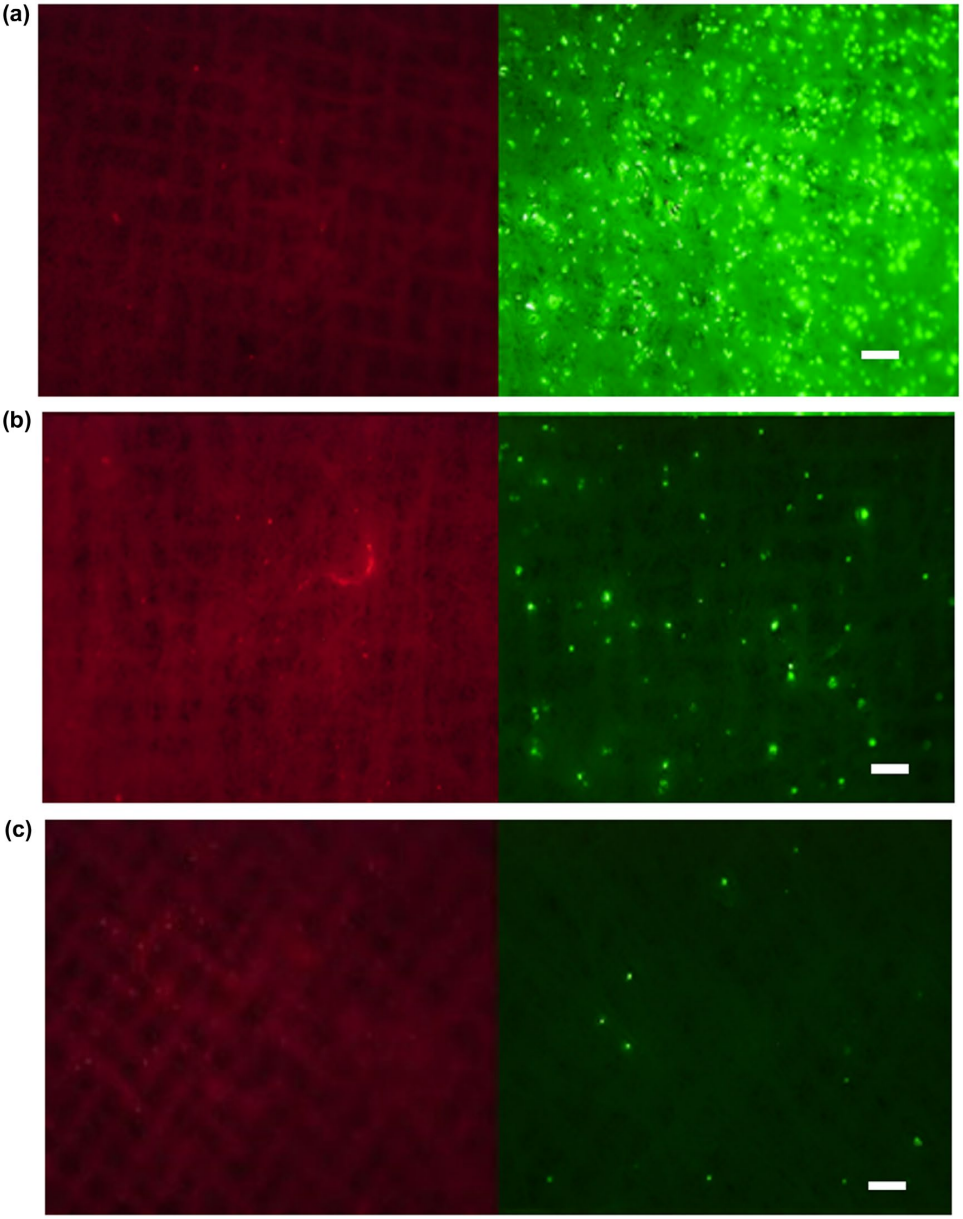
Capturing efficiency of MCF-7 cells from the cell mixture with CCRF-CEM cells. PS microfiber fabrics were observed (left: CCRF-CEM cells, right: MCF-7 cells) after the cell mixture of MCF-7 cells and CCRF-CEM cells was passed through the microfiber fabric with the cell number of (a) 1 × 10^5^ cells and 1 × 10^5^ cells, (b) 1 × 10^4^ cells and 1 × 10^5^ cells, and (c) 1 × 10^3^ cells and 1 × 10^5^ cells, respectively. Scale bars: 250 μm.

**Figure 6.**
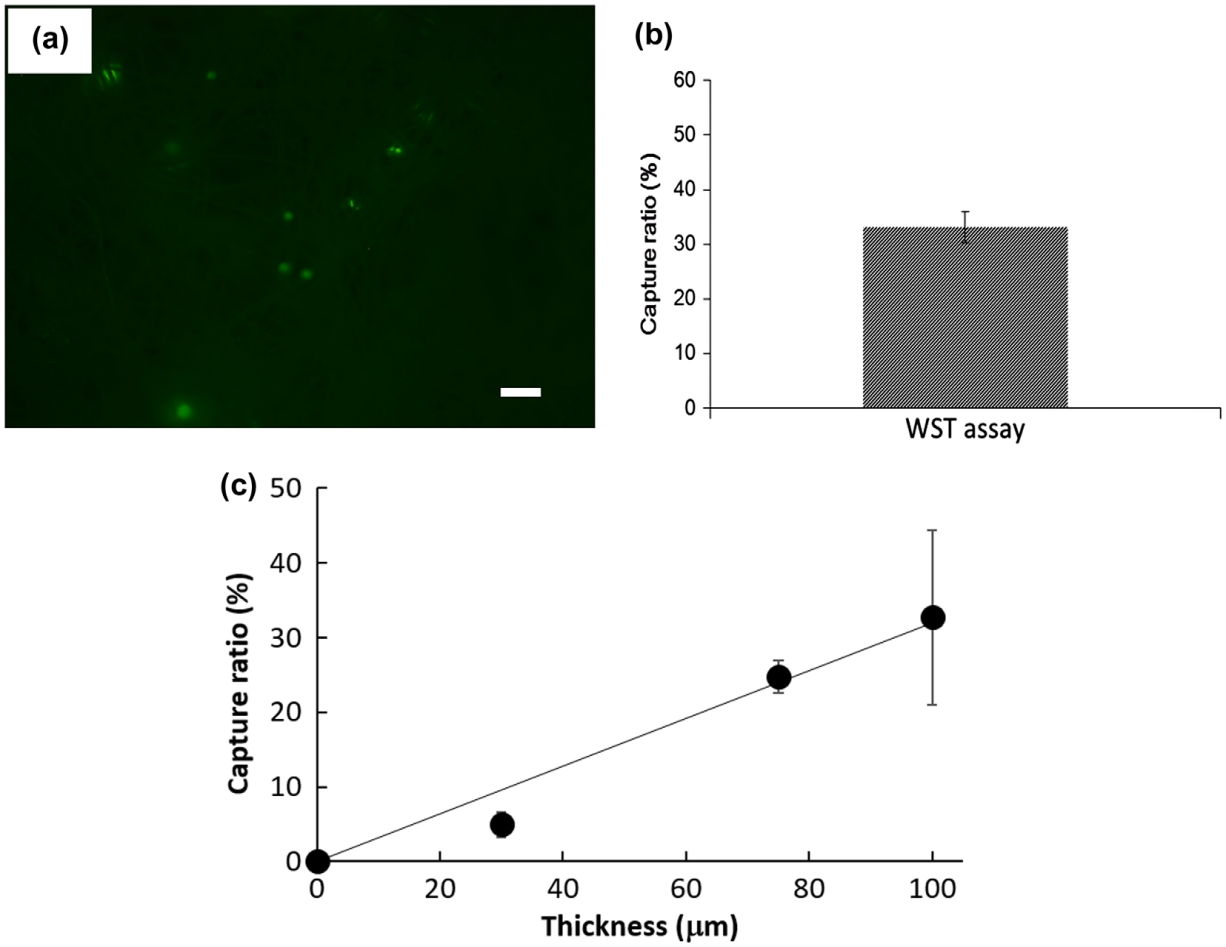
(a) Pig whole blood spiked with MCF-7 cells was passed through the microfiber fabric. Scale bar: 100 μm. (b) Cell capture ratio of electrospun PS microfiber fabric with 100 μm thickness. (c) Relationship between cell capture ratio and thickness of the electrospun PS microfiber fabric is shown (*n* = 3). Results are presented as means ± SD.

We then examined the selectivity of the cell capturing process when cells were mixed with other cells, since there are lots of different blood cells in whole blood, e.g. erythrocytes, leukocytes and platelets. After MCF-7 cells were mixed with CCRF-CEM cells, a floating cell line, the mixture was applied to the PS microfiber fabric at the same flow rate as before (Figure 5(a–c)). Here, the cell numbers of MCF-7 cells and CCRF-CEM cells were 1 × 10^5^ cells and 1 × 10^5^ cells, 1 × 10^4^ cells and 1 × 10^5^ cells, and 1 × 10^3^ cells and 1 × 10^5^ cells, respectively. MCF-7 and CCRF-CEM cells were fluorescently labeled in advance with green and red fluorescence, respectively. MCF-7 cells were successfully captured on the fabric, while CCRF-CEM cells were not, indicating the selective capturing of MCF-7 cells from the mixed cell population. The capturing efficiency depended on the existing MCF-7 cell numbers. The capturing selectivity was examined using whole blood spiked with MCF-7 cells. We also examined the capturing ability in blood mixed with MCF-7 cells. The mixed blood was applied to the PS microfiber fabric as in previous experiments. Captured MCF-7 cells could be observed on the PS microfiber fabric (Figure 6(a)). From these results, our designed system could specifically capture EpCAM-positive cells within seconds from other impurities. Next, the cell capture ratio was evaluated when changing the PS microfiber fabric thickness that was controlled by the electrospinning time. For quantitative analysis of the number of cells captured on the PS microfiber fabric, we used the WST assay. The result is shown in Figure 6(b). When the capture ratio was calculated from the number of cells before and after vacuum aspiration with the WST assay, it was 33 ± 3% for the fabric thickness of 100 μm. The capture ratio was significantly improved by the increase of fabric thickness (Figure 6(c)). In our systems, the targeted cells were specifically captured on PS microfiber fabric when the thickness was between 30 and 100 μm. Therefore, with increased electrospinning time, total contact surface area could also be increased, which results in the improvement of the cell capturing capacity. When compared to the conventional leukocyte size separation from whole blood using polymeric microfibers,[22,24] blood cells were not entrapped onto the PS microfiber fabric in our system. The CTCs would be specifically captured by the interaction with immobilized antibody.

In our vacuum aspiration approach, it is possible to directly apply whole blood to electrospun PS microfiber fabric without any pre-treatment of cells such as labeling with antibodies, and we do not need to dilute the whole blood. The hemolysis ratio was less than 10% for our system. Since the pore size and diameter of PS microfiber fabric were optimized, there was less hemolysis after the whole blood was passed through the PS microfiber fabric. In addition, total analysis time would be significantly shortened compared to the other existing methods. However, we need to improve our approach for further analysis. Since the number of CTCs in the blood stream is small (1–100 cells in 1 ml), ideally it should be possible to collect CTCs with higher accuracy. With further improvement, this anti-EpCAM-antibody-immobilized microfiber fabric system with vacuum aspiration would allow for an unprecedented, ultrafast cell isolation method, and thus be suitable for quick tumor diagnoses at clinical sites.

## 4. Conclusions

We fabricated three-dimensional electrospun PS microfiber fabrics to which anti-EpCAM antibody could be immobilized with high density. All of the whole blood could be passed through the microfiber fabric system by vacuum aspiration without damaging the blood cells. The combination of vacuum aspiration and anti-EpCAM-antibody-immobilized PS microfiber fabric could specifically capture EpCAM-expressing cells within seconds. The electrospun PS microfiber fabric device for rapidly capturing cells would be suitable for clinical, on-site applications.

## Disclosure statement

The authors declare that there are no conflicts of interest.

## Funding

This work was supported by the Ministry of Education, Culture, Sports, Science and Technology of Japan [24651158].
